# Filamin A-Hinge Region 1-EGFP: A Novel Tool for Tracking the Cellular Functions of Filamin A in Real-Time

**DOI:** 10.1371/journal.pone.0040864

**Published:** 2012-08-01

**Authors:** Jesús Planagumà, Laura Minsaas, Mónica Pons, Lene Myhren, Georgina Garrido, Anna M. Aragay

**Affiliations:** 1 Department of Biomedicine, University of Bergen, Bergen, Norway; 2 Institut de Biologia Molecular de Barcelona, CSIC, Barcelona, Spain; Fundação Oswaldo Cruz, Brazil

## Abstract

**Background:**

Filamin A (FLNa) is an actin-crosslinking protein necessary for stabilizing the cell surface, organizing protrusive activity and for promoting efficient cellular translocation. Recently, our group demonstrated the requirement of FLNa for the internalization of the chemokine receptor CCR2B.

**Methodology and Principal Findings:**

In order to study the role of FLNa *in vitro* and in real-time, we have developed a fluorescent FLNa-EGFP construct. In this novel imaging tool, we introduced the EGFP-tag inside the flexible hinge 1 region of FLNa between two calpain cleavage sites. Our findings indicate that the FLNa-EGFP construct was correctly expressed, cleaved by calpain and colocalized with actin filaments as shown by immunostaining experiments in the human melanoma cell lines A7 (FLNa-repleted) and M2 (FLNa-deficient). In addition, scanning-electron microscopy (SEM) and micropatterning studies also provided clear evidence that the cell rigidity was restored. FLNa-EGFP allowed us to demonstrate the interaction of FLNa with the chemokine receptor CCR2B in endocytic vesicles after CCL2 ligand stimulation. Through live-cell imaging studies we show that the CCR2B receptor in Rab5-positive vesicles moves along filamin A-positive fibers.

**Significance:**

Taken together, these results outline the functionality of the FLNa-EGFP and the importance of filamin A for receptor internalization and movement into endocytic vesicles.

## Introduction

Filamin A (FLNa), previously known as ABP-280, is the most widely expressed and most potent actin filament-crosslinker in a family of actin-binding proteins (ABP) and is critically involved in both locomotion and cell rigidity [1]. Structurally, FLNa is comprised of three defined structures: an N-terminal actin-binding domain (ABD), a rod-shaped domain composed of twenty-four tandem immunoglobulin (Ig)-like repeats of ∼96 amino acids each [1], [2], [3] and two hinge regions, which each contain a cleavage site for the calcium-dependent protease calpain. Hinge 1 (H1) connects repeats 15–16 and Hinge 2 (H2) connects repeats 23–24, providing filamins with their characteristic V-shaped flexible structure [2], [4]. The H1 region also divides the rod-shaped domain into two subdomains, Rod 1 (repeats 1–15) and Rod 2 (repeats 16–24). The N-terminal ABD consists of two calponin homology (CH1 and CH2) domains containing three principal actin-binding sites [5], [6], while the 24^th^ C-terminal Ig-like domain is the dimerization domain, which is crucial for the actin-crosslinking function of filamins [7].

Filamins also have other functions besides their F-actin crosslinking ability. In fact, FLNa has over sixty reported intracellular interaction partners, including membrane receptors, signalling intermediates, enzymes, ion channels, and transcription factors [8], [9]. The diversity of the binding partners of the FLNa protein makes it difficult in principle to evaluate the physiological significance of those interactions as a whole. Nevertheless, when the interaction partners are classified, it becomes apparent that a vast majority of these include membrane proteins like the β-integrins [10], [11], [12] and the extracellular calcium receptor [13], [14], signalling proteins such as the family of small GTPases Rac, Rho and Cdc42 [15], or transcription factors like the androgen receptor [16]. This observation suggests that FLNa provides mechanical stability to the cell membrane and maintains cell-cell and cell-matrix connections by tethering membrane receptors to the actin cytoskeleton. In addition, FLNa may play an important role as a scaffold by facilitating and coordinating cellular processes, especially those involved in actin polymerization.

The vast majority of the proteins that bind to FLNa interact with the Rod 2 domain of the protein [9]. This is also the case for the CC-chemokine receptor CCR2B, which we recently identified as a direct interaction partner of FLNa. According to our findings, FLNa is required for the efficient ligand-induced internalization of the receptor and possibly also for other downstream processes involving the receptor [17]. Interestingly, the CCR2B receptor is not the only chemokine receptor that binds to FLNa. The CCR5 and CXCR4 receptors have also been found as partners for filamin, though they interact with different repeat structures of the FLNa protein [18]. Additionally, CD4 also interacts with FLNa [18] and CCR5, which then functions as a structural adaptor for CD4 and chemokine receptor clustering in T cells. On the other hand, CD28 requires FLNa binding to induce T-cell cytoskeletal rearrangements in the immunological synapse [19]. So FLNa appears to be particularly important for the physiological responses induced by chemokine receptors and for the immunological responses. Moreover, a growing number of other GPCRs have lately been associated with this actin-binding protein, suggesting new physiological responses in which FLNa might play an important role.

In order to obtain a molecular tool that would help to study the physiological functions of FLNa, we have developed a fluorescent FLNa fusion construct, which could be used for visualizing its dynamics employing live-cell imaging. As the N-terminal domain of FLNa is critical for actin-binding and the C-terminal domain is involved in dimerization and binding to multiple intracellular interaction partners, we chose to place the EGFP-tag in the flexible H1 region, to avoid blocking any of these important physiological functions of FLNa. Our findings indicate that the FLNa-EGFP construct was correctly expressed and colocalized with actin filaments, providing rigidity to the cells. Furthermore, the fusion protein was able to bind and internalize the chemokine receptor CCR2B upon CCL2-stimulation in Rab5-positive vesicles. The ligand-stimulated ß2-adrenergic receptor also internalized along FLNa-positive vesicles at early times. In summary, FLNa-EGFP is a novel imaging tool that will help to understand its multiple functions in the cell, and especially to follow the internalization of membrane receptors.

## Results

### FLNa-EGFP was successfully expressed in cells and cleaved by calpain

**Figure 1:**
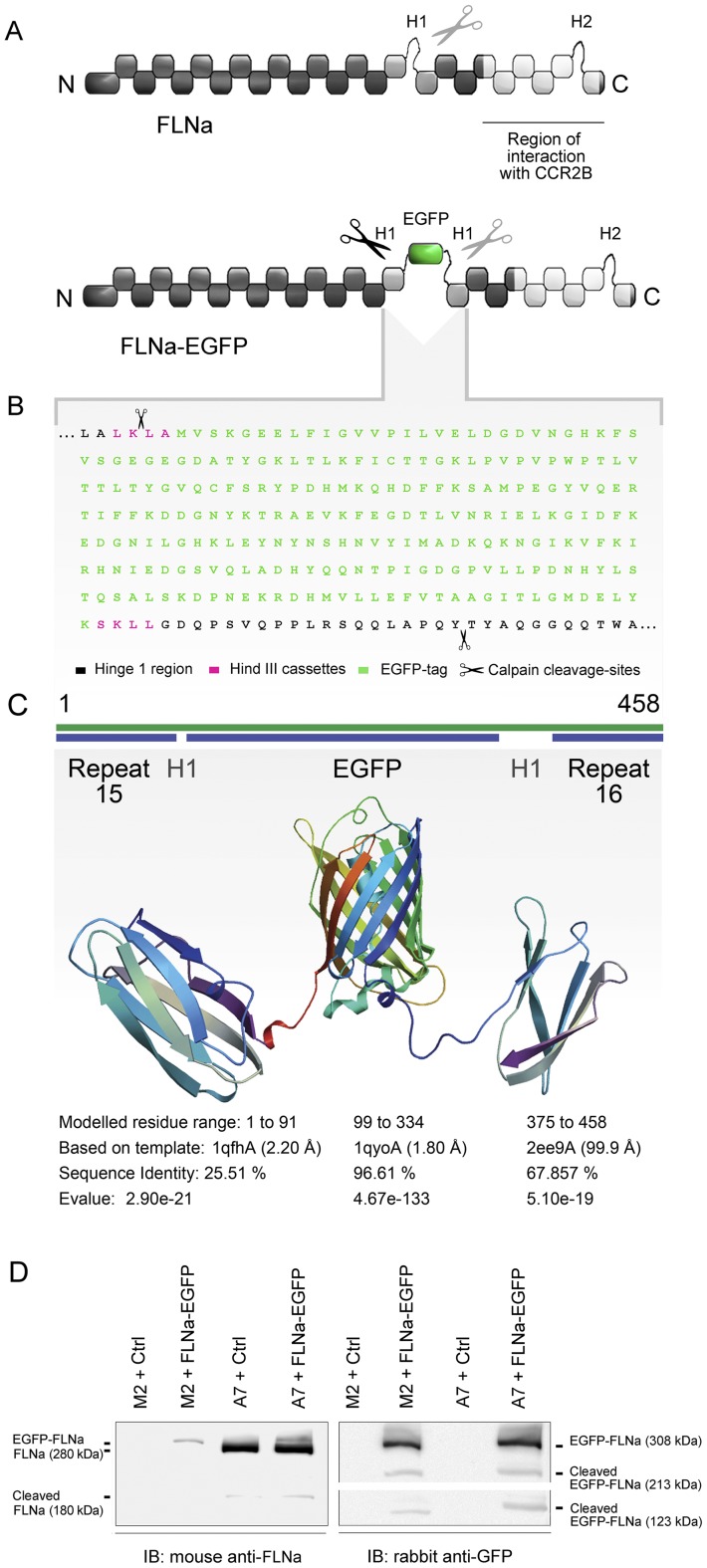
Structure of the FLNa-EGFP construct. (**A**) Schematic representation of the structures of wild-type human FLNa (upper drawing) and the FLNa-EGFP fusion construct (lower drawing). The region of interaction with CCR2B and the site of insertion of the EGFP-tag in the Hinge 1 region (H1) are indicated. (**B**) Amino acid sequence of the hinge 1 region of FLNa-EGFP. The EGFP-tag is flanked by two Hind III cassettes (pink). Scissors point to the natural (in the Hinge 1) and artificially inserted (in the first Hind III cassette) calpain-cleavage sites. (**C**) Homology modeling prediction of the fragment comprising FLNa repeats 15 and 16 flanking the hinge 1 region with EGFP integrated in the middle. Modeling was accomplished using the SWISS-model server. (**D**) Immunoblots showing the expression of FLNa-EGFP. A7 and M2 cells were transiently transfected with pcDNA3.1-FLNa-EGFP (+FLNa-EGFP) or empty pcDNA3.1 (Ctrl). Blots were probed with anti-FLNa or anti-GFP antibodies as indicated. Black lines indicate the migration bands of full-length monomeric FLNa-EGFP, endogenous FLNa and calpain-cleaved FLNa and FLNa-EGFP. Blots are representative of at least three independent experiments.

Filamin A is involved in a multitude of cellular processes such as cell migration, protein trafficking, transcriptional regulation and mitosis [9]. The ability of FLNa to crosslink actin filaments, to dimerize, and to interact with intracellular partners is crucial for theses processes. In our quest to design an *in vitro* fluorescent FLNa imaging tool, it was thus important to choose a cloning strategy that would prevent compromising any of its physiological functions. We therefore selected the flexible hinge 1 region as a target for the insertion of a fluorescent EGFP tag. The hinge 1 region harbours one of the two calpain-cleavage sites in FLNa, the other being located 10 kDa from the carboxy-terminal end in the hinge 2 region [1]. To avoid masking the calpain cleavage site of FLNa, which among other things is important for the solation of focal adhesions at the uropod during cell migration [20], [21], we flanked the EGFP-tag with two small linker regions, and placed the tag close to the beginning of the H1 sequence (Figure 1B). To ensure that the FLNa-EGFP protein would be cleaved in the H1 region, we also introduced an artificial calpain-cleavage site in the linker region in front of the EGFP-tag (Figure 1A and B). As can be observed in the EGFP-tag model (Figure 1C), the insertion of EGFP in the flexible H1 region between repeats 15 and 16, would probably not compromise the structure of the FLNa protein (Figure 1C). Western blot analyses from A7 (FLNa-repleted) and M2 (FLNa-deficient) cells, transiently transfected with the FLNa-EGFP or an empty vector, revealed that the FLNa-EGFP fusion protein was expressed and migrated at its predicted size (Figure 1D, upper bands in both panels), just above the endogenous FLNa band (left panel). The construct could be detected using antibodies targeted against FLNa (left panel) and GFP (right panel), confirming the in-frame insertion of EGFP in the hinge 1 region. Interestingly, the FLNa-EGFP construct was cleaved by calpain either before (right panel, lower band) or after (right panel, middle band) the EGFP-tag. The naturally cleaved calpain-band of endogenous FLNa can be seen in the left panel of Figure 1D. So the results indicated that the chimeric protein was expressed and that the insertion of EGFP does not interfere with the normal calpain-cleavage of FLNa in the H1 region.

### FLNa-EGFP colocalizes with actin and is present in the same subcellular locations as endogenous FLNa

**Figure 2:**
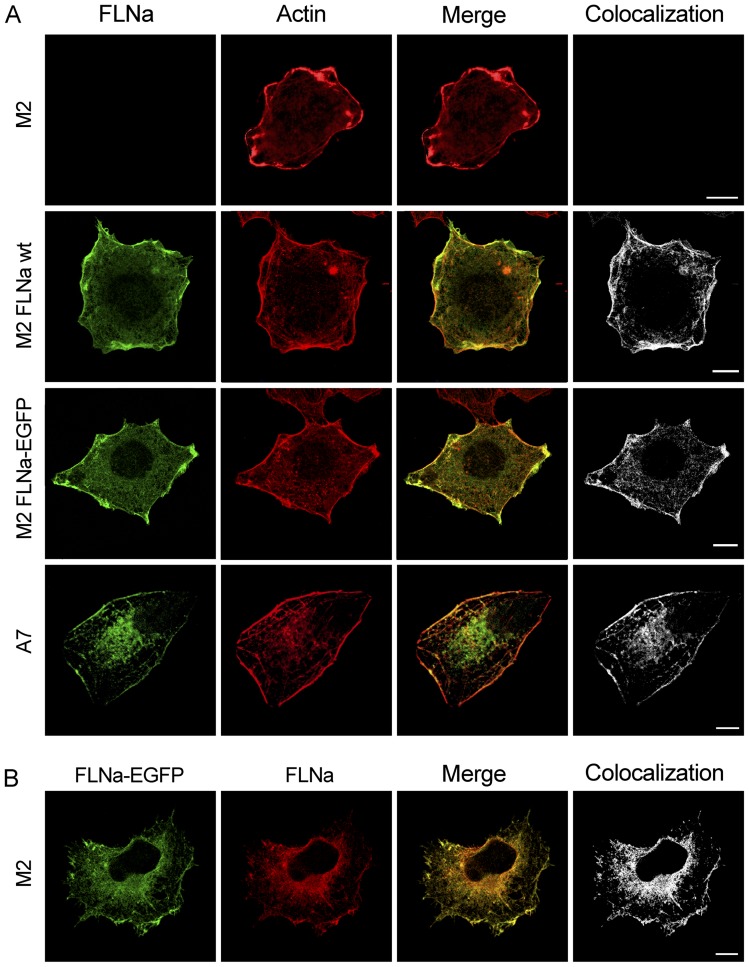
FLNa-EGFP colocalizes with actin at the membrane and in fibres. (**A**) Confocal images of FLNa, FLNa-EGFP and actin expression in cells after immunostaining. M2 cells transiently transfected with pcDNA3.1-FLNa-EGFP or pREP4-FLNa and untransfected A7 and M2 cells, were seeded on top of glass coverslips, fixed, permeabilized, incubated with anti-FLNa and goat anti-mouse Alexa Fluor 488 and stained with rhodamine-phalloidin before mounting. (**B**) Confocal images of FLNa-EGFP expression in M2 cells transiently transfected with pcDNA3.1-FLNa-EGFP. Cells were seeded on top of glass coverslips, fixed, permeabilized and incubated with anti-FLNa and Texas Red goat anti-mouse. All images are from one single layer of the Z stacks. The colocalization between (green) and (red) was analyzed using Imaris colocalization software and is shown in white. Images are representative of at least three independent experiments. Scalebars (10 μm).

Next it was important to assess the functionality of the FLNa-EGFP protein. As mentioned before, FLNa is an actin-crosslinking protein which colocalizes with actin structures in the cell. So, immunofluorescence experiments could give us the first clues of the performance of this chimeric protein. Again we took the advantage of expressing the chimeric fluorescent protein in the filamin-depleted M2 cells. First, M2 cells were transiently transfected with either FLNa-EGFP (Figure 2A, third row of panels) or wild type FLNa (second row of panels), and cells were stained for actin with Rhodamine-phalloidin. The images show a clear colocalization of EGFP- and endogenous filamin A with actin (see merged image and colocalization) indicating that FLNa-EGFP displayed the same subcellular localization pattern as endogenous FLNa in A7 cells (lower panels). Moreover, staining M2-FLNa-EGFP cells with anti-FLNa antibody resulted in a perfect colocalization of both fluorescent labels, which again supports the idea that the chimeric protein is correctly expressed and detectable with anti-FLNa antibodies (Figure 2B). Untransfected M2 cells were as expected devoid of FLNa (upper panels). This confirms the first indication that the actin-binding function of FLNa-EGFP is preserved and that it has not been compromised by the fluorescent tag.

### A7-like morphology is restored in cells expressing FLNa-EGFP

**Figure 3:**
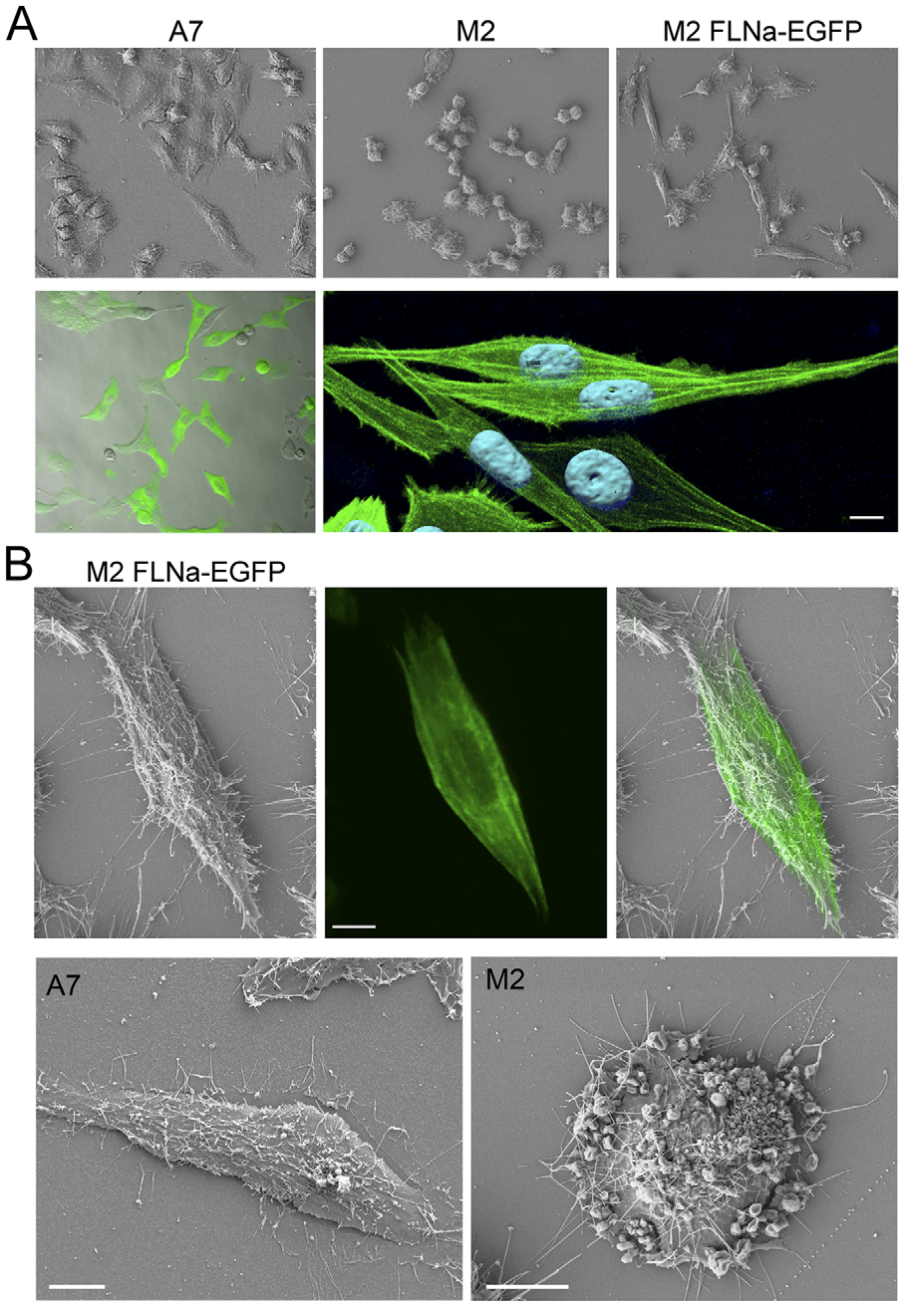
FLNa-EGFP restores the A7-like morphology in M2 cells. (**A**) (Upper panels) SEM images of A7 and M2 cells and mixed M2 FLNa-EGFP cell population (magnification 40x). (Lower panels, left) Fluorescent image combined with bright-field image of M2 cells expressing FLNa-EGFP (magnification 40x). (Lower panels, right) Confocal microscopy image of M2 cells expressing FLNa-EGFP processed with Imaris (scalebar: 10 μm). (**B**) (Upper panels) SEM and fluorescent correlation microscopy: Image from the same cell taken by SEM and fluorescent microscopy and overlapped with adobe photoshop (scalebars: 10 μm). (Lower panels) SEM images of A7 and M2 cells (scalebar: 10 μm).

While the FLNa-repleted A7 cells are dispersed before growing to confluency and characterized by an elongated morphology with the occurrence of surface stress fibres, FLNa-deficient M2 cells are known for growing in cohesive colonies, for their lack of actin fibre bundles and for their extensive membrane blebbing due to cell surface instability [22], [23]. In order to get a more detailed picture of the structural consequences of introducing FLNa-EGFP into FLNa-deficient cells, we used scanning electron microscopy (SEM) in combination with fluorescence microscopy. High-resolution SEM and fluorescent images revealed that the morphology of FLNa-EGFP-expressing cells (Figure 3A, upper right and bottom panels) was clearly A7-like (Figure 3A and B), displaying typically elongated cells with stress fibres and a clear reduction in surface blebbing compared to the FLNa-deficient M2 cells (Figure 3B). Also worth noticing, was the perfect correspondence between FLNa staining and the stress fibre structures (Figure 3B, upper right panel). These findings suggest again that FLNa-EGFP is able to crosslink actin like the wild-type FLNa.

**Figure 4:**
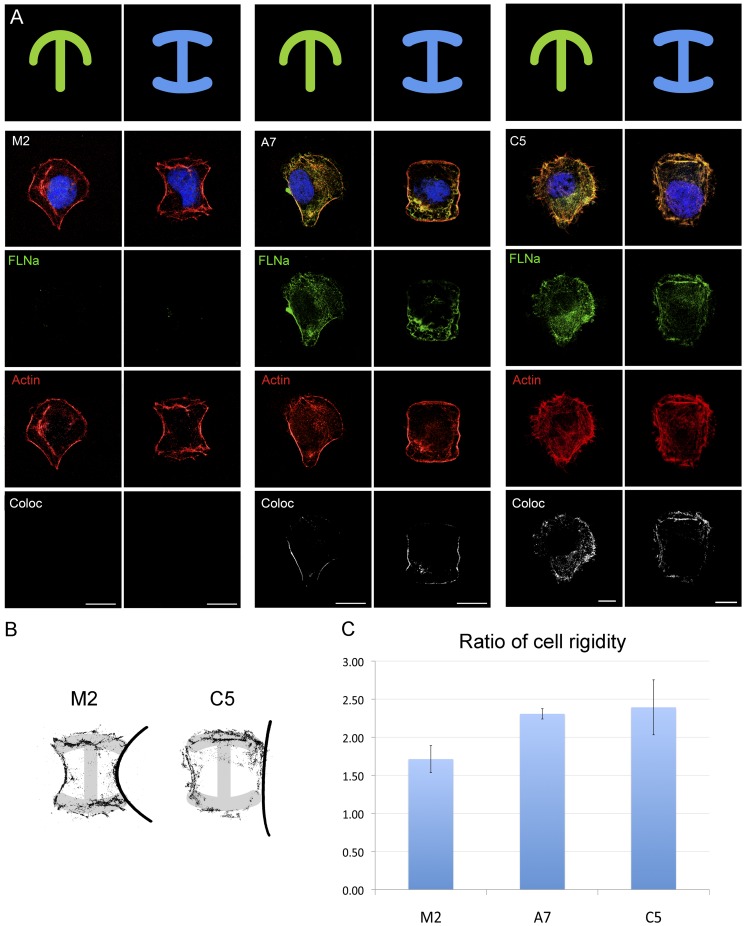
Cell rigidity restored by FLNa-EGFP. (**A**) Micropatterning assay showing two fibronectin shape types (T and I) covered by M2, A7 and C5 (clone with FLNa levels resembling those of A7 cells). Images taken by confocal microscopy are from one single layer of the Z stacks. FLNa-EGFP and FLNa are shown in green, actin in red and the colocalization channel, calculated with the Imaris software, in white (scalebars: 10 μm). (**B**) Detail of M2 and C5 cells showing the actin fibre curvature over the micropattern shape. (**C**) The size of the area in pixels of the A7, M2, and C5 cells attached to the pattern was divided by the area of the fibronectin shape and given as ratio (area cell/area fibronectin) (Ratio close to 1 =  area covered by cell is equal to the area covered by fibronectin =  low cell rigidity). The experiment was repeated three times and 30 cells were analyzed per point. Results were plotted in a chart with (± SEM).

From the mixed population of M2 cells stably expressing the construct, we isolated a population of cells expressing medium and high levels of fluorescence using flow cytometry. This population was then serially diluted in order to isolate individual clones. Careful observations of the cells using regular light-microscopy and fluorescence microscopy (Figure S1 A), in combination with analyses of cell lysates (Figure S1 B), showed that clones with lower levels of fluorescence and protein expression behaved more M2-like, displaying more blebbing and a tendency to grow in cohesive colonies (data not shown). On the other hand, cells with too high levels of FLNa, like C4, showed a tendency to grow in circular patterns. Cells that had FLNa expression levels similar to the levels found in A7 cells had a more elongated morphology (e.g. clones 5 (C5) and 8 (C8)) (Figure S1 A and B). Clone 8 had many elongated cells with a high occurrence of surface stress fibres, but the cells in this clone also seemed to actively round up and cluster and did not grow as fast as the A7 cells. These results show the importance of the levels of FLNa for the viability and functionality of the cell.

### Cell rigidity in the clonal cells is restored to the levels of A7 cells

It has recently been shown that due to their lack of FLNa, M2 cells are twice as soft as, more fluid-like, and exert much smaller contractile stress on the substratum than the more rigid FLNa-expressing A7 cells [24]. To determine if FLNa-EGFP could restore the rigidity in M2 cells, we chose to compare the structural behavior of A7, M2 and cell clone C5 using the new fibronectin micropatterning technology, recently commercialized by CYTOO. Clone C5 had similar levels of FLNa expression and similar morphology compared to A7 cells. So we used this clone for cell rigidity analyses. The cell shaped images showed that M2 cells were able to draw well-defined T and I shapes compared to the A7 cells and clone C5 (Figure 4A). To quantify the rigidity of the cells, the measurement of the area (in pixels) of the cell attached to the pattern was taken and divided by the area of the fibronectin pattern. A ratio equal to one would mean a perfect overlap with the fibronectin pattern, indicating lower structural rigidity (Figure 4C). Cells without FLNa (M2) seemed much more plastic and adaptable to a given shape than their partners with FLNa (A7 and M2-FLNa-EGFP) (Figure 4B). In fact, the cells expressing FLNa had a common characteristic of drawing poorly defined T and I shapes, revealing that the more rigid architecture of these cells was due to the presence of FLNa. When comparing A7 vs. C5 cell shapes, the phenotype of C5 resembled the one of A7. These observations suggest that the levels of FLNa are important for the correct establishment of the FLNa-EGFP clones. We also used the micropatterns to study the cellular localization of filamin A and actin. Interestingly, images revealed an intensive colocalization of actin and FLNa in the boundaries of the cell, especially in leading edge, indicating hot spots for structural reorganization and directional migration.

### Migration speed of M2 cells is enhanced in cells expressing appropriate levels of FLNa-EGFP

As FLNa-deficient cells are known to have impaired translational migration [23], we sought to investigate if FLNa-EGFP could also restore this function. Time-lapse studies following the migration of cells over time (Figure S2, lower panels) were first acquired with a mixed population of M2-FLNa-EGFP stable cells. A7 (middle panels) and M2 (upper panels) cells were followed as positive and negative controls, respectively. The average M2 cell (without FLNa) showed a reduced capacity to move on the plate, as has previously been reported [23]. On the contrary, most of the A7 cells were moving. Figure S2 shows an example of the movement of a single A7 cell on the plate. M2-FLNa-EGFP cells that had a more elongated phenotype were also able to migrate similarly to the A7 cells.

**Figure 5:**
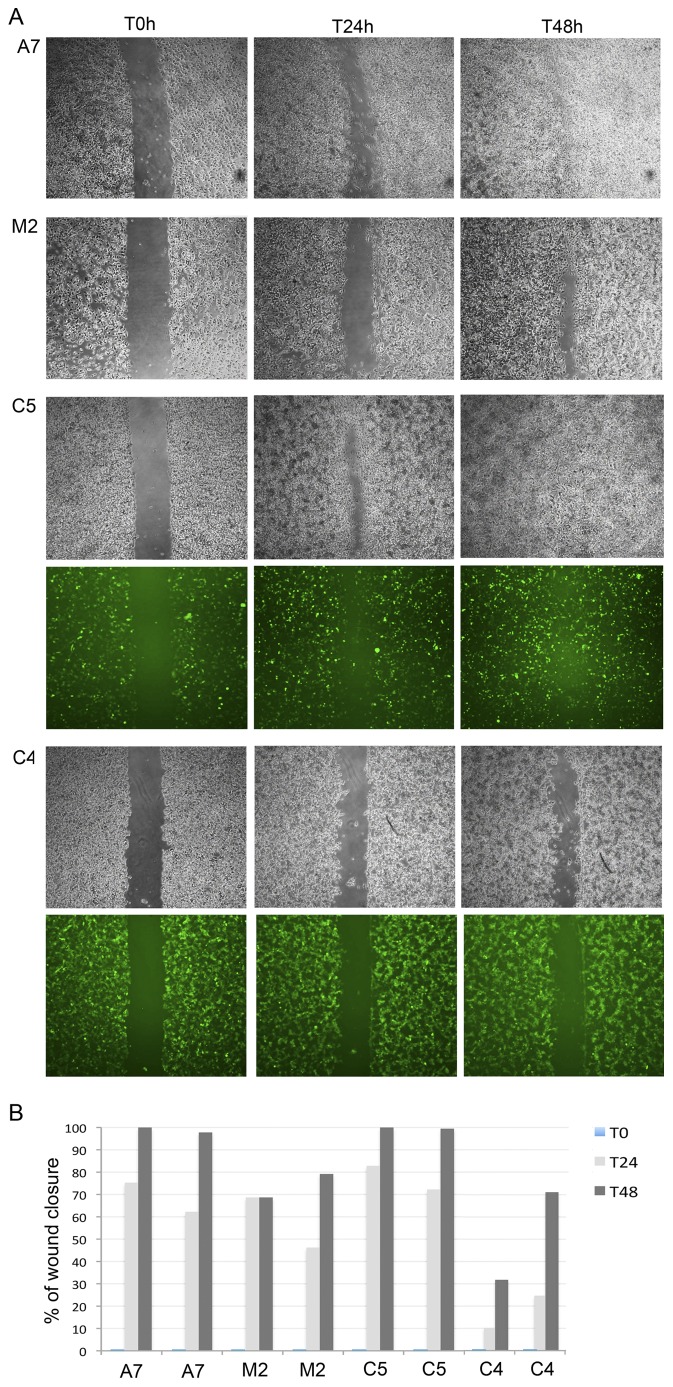
FLNa-EGFP restores wound healing velocity of M2 cells. (**A**) Brightfield and immunofluorescent images of wound closure in different cell lines. Confluent monolayers of A7, M2 and selected M2-EGFP-FLNa cell clones (C5 and C4) were scratched with a pipette tip and images were taken at 0, 24, and 48 h after wounding, as indicated. Images were taken with 4× magnification using a PhL filter. Experiments were done in duplicates and repeated three times. (**B**) ImageJ quantification of the wound-healing assay analysing the area of the wound for each time point and cell type (A7, M2, C5 and C4). Results plotted as a % of the wound closure per time point.

We then studied the effect that the different expression levels of FLNa-EGFP would have on cell migration by using two clonal cell lines C4 and C5, expressing different filamin A levels, in a wound-healing assay. As mentioned before, C5 had similar levels of FLNa as the A7 cells. Confluent A7 and M2 cells and stable cell clones 4 (C4) and 5 (C5) were wounded and allowed to migrate into the cleared area (Figure 5A). Employing ImageJ software, the rate of wound closure was analysed determining the size of the area of the wound for each cell line and time point (Figure 5B). Untransfected M2 cells covered ∼57.5% of the wound area after 24 h and ∼74% after 48 h, while wild-type A7 cells and clone 5 covered ∼68% and ∼77% wound closure after 24 h and showed near complete wound closure after 48 h (A7: ∼99% and C5: ∼99%). Interestingly, the high FLNa-EGFP level expressing clone 4 performed even worse than the M2 cells showing only ∼17% wound closure after 24 h and ∼51% wound closure after 48 h (Figure 5A and B) As mentioned before, the high levels of FLNa induces the cells to grow in circular patterns. These results suggest that overexpression of FLNa-EGFP decreases cell locomotion, while expression of the appropriate levels of FLNa-EGFP restores the migration speed to the level of A7 cells, implying that the chimeric protein is functional when expressed at the adequate levels. These results also corroborate the essential role that this actin-crosslinking protein has on directionally migration and that the EGFP fusion construct is a useful tool for visualizing these processes.

### Efficient CCL2-mediated internalization of CCR2B is restored using the fusion construct

**Figure 6:**
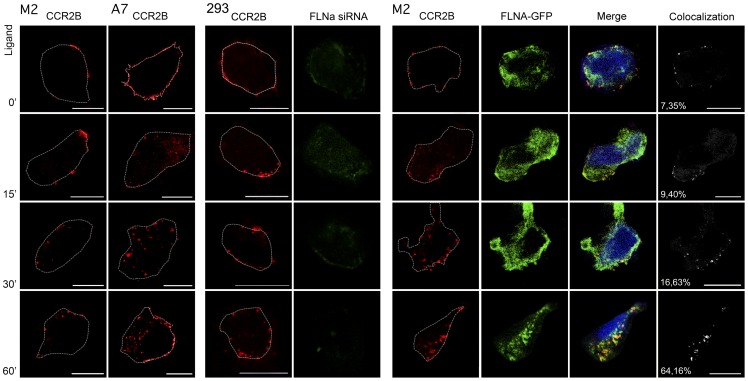
FLNa-EGFP binds and internalizes CCR2B. M2 cells (first column left panel), A7 cells (second column left panel) and HEK293 cells were transfected with pcDNA3-FLAG-CCR2B. Central panel shows HEK293-CCR2B cells treated with 100 nM of FLNa siRNA. M2 cells were transfected with pcDNA3-FLAG-CCR2B and FLNa-EGFP (right panel). Cells were further incubated with CCL2 for the times indicated and stained for CCR2B (red). The images were deconvolved using a theoretical point spread function before analysis of colocalization. Colocalization is shown in white with Pearson coefficient expressed in %. Images are from one single layer of the Z stacks. White dotted lines show the contour of the cells. Experiments were done in duplicates and repeated three times in each experiment an average of 20 cells were analyzed. Scalebars (10 μm).

**Figure 7:**
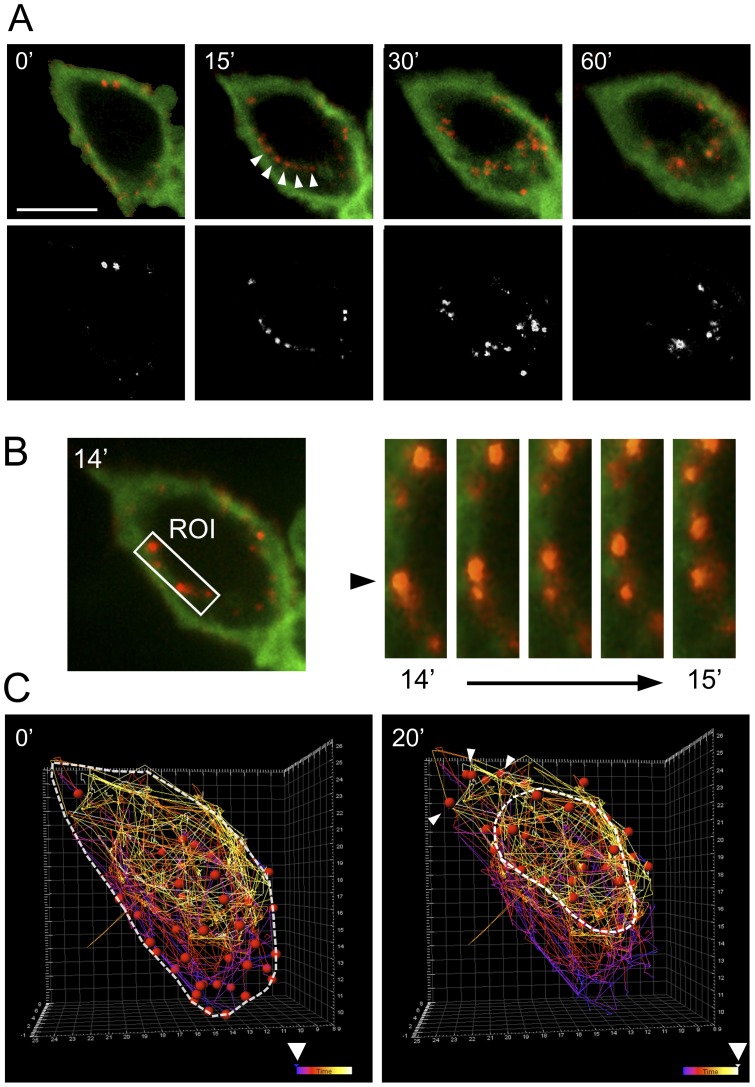
Time lapse of the Internalization of the CCL2-stimulated CCR2B in HEK293 cells expressing FLNa-EGFP. (**A**) Upper row: snapshots from spinning disk experiments with HEK293 cells showing the distribution of CCR2B (red) and FLNa-EGFP (green) in different time points. Arrow depicts vesicles located to filamin A-containing fibers. Lower row: colocalization analysis shown in white. (**B**) ROI analysis of the CCRB internalization at the time interval from 14 to 15 min showing the movement along the stress fibers. (**C**) Tracking analysis using Imaris software shows the CCR2B trajectory (red balls) after CCL2 stimulation from time 0 min (left) until time 20 min (right). Tracking lines are color-coded ranging from purple (0 min) until yellow (20 min). Arrow depicts the leading edge of the cell. Scalebars (10 µm).

Previous work from our group has demonstrated that FLNa binds to the C-terminal intracellular tail of the chemokine receptor CCR2B [17]. In order to track the internalized receptor and filamin A, experiments were designed in FLNa-deficient M2 cells expressing the CCR2B receptor and FLNa-EGFP and FLNa siRNA-treated HEK293 cells (Figure 6). We used CCR2B-expressing A7 and M2 cells as control. There was poor internalization of the receptor, in absence of FLNa after 15 and 30 min of ligand incubation in M2 cells and complete inhibition of internalizaion in FLNa siRNA-treated cells that express low levels (20%) of FLNa (Fig S3). However, in A7 and FLNa-EGFP-repleted M2 cells, most of the receptor was internalized after 15 and 30 min of CCL2 challenge (Figure 6). This can be clearly seen following the percentage of co-localization (Pearson coefficient, expressed in %) which increased with the time after ligand addition reaching values of 64% after one hour. So these results demonstrate that FLNa-EGFP is necessary for the internalization of ligand-induced CCR2B receptor. Then, experiments were designed to follow the receptor after CCL2 stimulation in presence of FLNa-EGFP *in vitro* and in real-time (Figure 7). For that, cells stably expressing the FLAG-tagged CCR2B receptor and transiently transfected with FLNa-EGFP were pre-incubated with the pre-labeled anti-FLAG antibody and then challenged with CCL2 under confocal microscopy (spinning disk) (Figure 7A). The receptor present at the plasma membrane moved inside the cells and co-localized with FLNa-EGFP containing fibers. Some of the vesicles containing the receptor can be seen moving along filamin-labeled fibers, which are likely to be actin fibers (Figure 7A arrows, 7B). Tracking of the particles (CCL2-stimulated CCR2B receptor) utilizing the Imaris software which depicts movements of particles with the time in a color coded diagram, allowed a better visualization of the trajectory of the receptor (Figure 7C). The particles represent the individual receptor internalized in endosomes. At early times particles are located at or close to the membrane (Figure 7C, violet color). A ring of particles moving around the nuclei is apparent in later times after ligand addition (Figure 7C, yellow-white colours, dotted line) which suggests that vesicles with internalized receptor were concentrating around the perinuclear region of the cell and that they did so by traveling along filamentous structures containing FLNa. Also, some particles moving at the edge of the cell appear at later times (Figure 7C, arrow).

**Figure 8:**
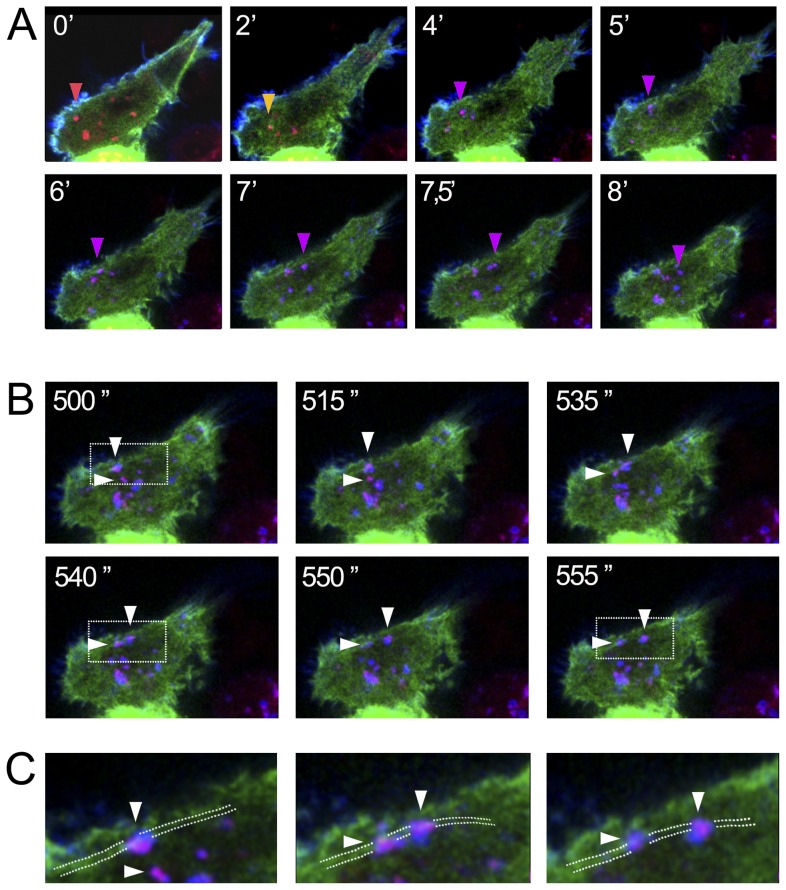
Time lapse of the Internalization of the CCL2-stimulated CCR2B in HEK293 cells expressing Cherry-Rab5 and FLNa-EGFP. HEK293 cells were transfected with pcDNA3-Flag-CCR2B, pCherry-Rab5 and pcDNA3.1-FLNa-EGFP. Cells were incubated with anti-flag M1 antibody followed by anti-mouse-Alexa647 antibody at 4°C. Cells were place in the 37°C microscopy chamber and stimulated with 20 nM CCL2. (**A**) Snapshots from Leica confocal SP5 microscope-time lapse experiments with CCL2-stimulated HEK293 cells (from 0 to 8 min) showing the distribution of CCR2B (blue), Cherry-Rab5 (red) and FLNa-EGFP (green) in different time points. Red arrow depicts the localization of the receptor at the plasma membrane; yellow are vesicles labeled with Cherry-Rab5 and FLNa-EGFP: violet are Rab5-vesicles containing CCL2-stimulated CCR2B, which are located to FLNa-containing fibers. (**B**) Snapshots of the CCRB internalization at the time interval from 500 to 555 s showing the movement of Rab5 and receptor positive vesicles along filamin A-containing fibers. Arrow heads indicate the position of two moving vesicles. (**C**) Detail analysis from B. Scale bars (10 µm). Dotted lines indicate the path of the filamin A possitive fiver.

To better assess if the internalized particles containing the ligand-stimulated CCR2B receptor were endosomal vesicles, cells were transfected with the receptor together with Cherry-Rab5 in presence if FLNa-EGFP (Figure 8, see also film in Fig S4). As can be seen, at early times the receptor (in blue) is localized at the plasma membrane together with FLNa (green) (Figure 8A), 4 min after ligand-activation the receptor internalizes and co-localizes at early endosomes with Cherry-Rab5-containing vesicles (red,+blue+green  =  violet) close to the cortex of the cell. It is possible to observe some of these vesicles containing the receptor and Rab5 moving along FLNa-containing structures (Figure 8A, 7 and 7.5 seconds. Figure 8B and C). These results then confirm that the internalized receptor in early endosomes moves along filamin containing fibers.

Since FLNa interacts with ß-arrestin2, which is involved in the process of internalization of other GPCRs like the ß2-adrenergic receptor ([25]), it is possible that this effect is not only observed with the CCR2B receptor. Experiments with time-lapse confocal microscopy were also performed with cells expressing the ß2-AR, together with Cherry-Rab5 and FLNa-EGFP (Figure S5). As expected, the ß2-AR internalized and localized to the early Rab5-labeled endosomes upon isopreterenol treatment. Early endosomes were also seen along some FLNa-positive fibers. Interestingly though ß2-AR-internalized vesicles localized with FLNa-EGFP only at very early time points (Figure S4, 03:56). These results agree with the fact the ß2-AR receptor dissociates from ß-arrestin2 at early times after internalization [26]. Together, these results support the notion that the binding to filamin facilitates the internalization of the ligand-stimulated GPCRs into endocytic vesicles.

## Discussion

In this paper, we have described a novel fluorescent FLNa tool and demonstrated its functionality and usefulness for visualizing the dynamic involvement of FLNa in various cellular processes employing live cell-imaging. The novelty of our tool arises from the strategic insertion of the fluorescent EGFP tag inside the flexible hinge 1 region of FLNa. More importantly, we demonstrated that the FLNa-EGFP protein restores the impaired internalization of the CCL2-stimulated CCR2B receptor in FLNa-deficient cells and demonstrated for the first time that filamin accompanies the receptor in endocytic vesicles, providing evidence for the involvement of actin structures in the internalization process of GPCRs.

In addition to crosslinking filamentous actin, FLNa also acts as an intracellular signalling scaffold for a large variety of membrane receptors and signalling molecules involved in cell adhesion and locomotion [1]. The list of protein partners that filamin A binds to, which so far includes over 60 members, is growing at an incredible speed, thus reflecting the impact of this protein on a wide range of cellular functions. So the design of a functional imaging tool to track FLNa would help to determine the role of this protein *in vitro* and in real-time. For that, it was crucial to choose a location for the EGFP-tag that would not compromise any of the important physiological function of FLNa. While the N-terminal ABD [5], [6] and Rod 1 [8] domains of FLNa are important for actin binding, the C-terminal domain is required for FLNa dimerization [7]. The dimerization of filamin is essential for its capacity to gelate actin filament structures. Also, the Rod 2 domain is the binding site for the vast majority of the intracellular interaction partners of FLNa [9]. The two hinge regions of filamin A show the greatest sequence divergence. Their main function is to provide flexibility and structural adaptability to the protein, though a recent study demonstrated that the hinge 2 region is also required for dimerization of FLNa, and for its interaction with the dimer of the Rac-specific FLNa-binding GTPase FilGAP [27]. The first hinge region between repeats 15 and 16 is mainly important for maintaining the viscoelastic properties of the actin networks. Actually, when a recombinant filamin lacking this hinge region was expressed, the mutant could still form actin networks, although they broke at lower stresses [28]. Here we have proven that the chimera protein is fully functional, which supports the idea that the hinge region 1 was structurally adequate for placing the EGFP. One of most striking differences between the M2 and A7 cells are their different capacities to form cell shapes on the micropattern fibronectin surfaces of the CYTOO arrays, due to their different adaptabilities. It has been proven that cells depleted of FLNa respond to mechanical forces [29]. Clearly FLNa-deficient cells adapted better to the shapes on the array than A7 cells or M2-FLNa-EGFP cells. Probably this effect is caused by the fact that under mechanical stress, FLNA-deficient cells elevate the levels of Rac whereas Rho activity decreases. In contrast, FLNa-expressing cells increase their Rho activity and number of stress fibres, which results is less adaptability to the T and I shapes.

Another challenge of placing the tag in this region was to avoid blocking calpain cleavage. Calpain cleavage is important for functional migration [20], [21] and for transcriptional regulation, as the cleaved 100 kDa C-terminal domain of FLNa has been found to localize to the nucleus with several transcription factors [16], [30], so to make sure that the fusion protein would be cleaved in this region, we also introduced and artificial calpain-cleavage site in front of the tag. It has been shown before that deletion of the calpain-cleavage site does not completely prevent calpain cleavage [31], so a potential obstruction of the natural calpain cleavage site could still lead to cleavage at another location. However, immunoblotting experiments indicated that both calpain-cleavage sites in the H1 region were equally functional and that FLNa-EGFP seemed to be cleaved either before or after the tag. Although the construct was partially cleaved in front of the EGFP-tag, we did not observe any FLNa expression in the nucleus in our preliminary experiments. This was probably due to low levels of the cleaved product, as only a small fraction of FLNa-EGFP is cleaved by calpain. Further research in different cell types and under various conditions utilizing FLNa-EGFP will help to study the role of the calpain cleavage in FLNa physiology.

Immunostaining experiments indicated that the FLNa-EGFP fusion protein colocalized with F-actin at the plasma membrane and in fibres as the wild type protein, implying that it was correctly expressed and able to interact with actin through its N-terminal ABD. In addition, we observed that FLNa-EGFP could restore translational migration in the FLNa-deficient M2. A combination of SEM and fluorescent microscopy allowed us to perform a detailed study of the morphology and cytoskeletal structures of M2 cells stably expressing the construct, revealing that FLNa-EGFP was also capable of cross-linking actin filaments as these cells had a high occurrence of surface stress fibres, decreased membrane blebbing and a more elongated phenotype, like the A7 cells [32]. One of the interesting observations of our research was the fact that the levels of expression of filamin A are crucial for obtaining a functional migrating cell. As so, both low and excessive levels of FLNa can inhibit cell migration [23]. Therefore, only few clones with moderate levels of filamin A expression behaved like A7 in cell cultures, growing dispersedly, displaying an elongated morphology and migrating similarly to the A7 cells. Excessive levels of FLNa-EGFP expression had a negative impact on cell growth and migration. The experiments evaluating cell rigidity and migration velocity during wound healing confirmed the need for adequate levels of filamin A for proper cell behaviour.

In a recent study from our group, we demonstrated that FLNa is required for the efficient internalization of the CC-chemokine receptor CCR2B, and that downregulation of FLNa in THP-1 monocytes prevented CCL2-mediated chemotaxis [17]. CCL2/CCR2B are involved in wide range of pathologies including multiple sclerosis, rheumatoid arthritis, atherosclerosis, cancer, and HIV-1 pathogenesis [33]. As mentioned before, we had previously demonstrated utilizing two-hybrid technology that the C-terminal tail of CCR2B binds to filamin through at least the repeat 19 domain. The interaction of FLNa with CCR2B appears to be necessary for the internalization step of the receptor into endocytic vesicles, but the results also seemed to indicate that further steps of vesicle trafficking could be involved. It was therefore necessary to confirm this novel role for filamin A in CCR2B trafficking. The results presented with the FLNa-EGFP clearly show that the internalized receptor localized with the FLNa-EGFP protein in a punctate pattern in the cytoplasm after internalization. So combined with our data demonstrating that knock-down of FLNa by RNA interference technology abrogates receptor internalization, these results demonstrate that filamin is present and necessary for the internalization and the trafficking of vesicles with the CCR2B receptor. Live cell imaging experiments enabled us to follow the dynamic involvement of FLNa and CCR2B during internalization. First, the movies showed that FLNa is an extremely motile protein, which is rapidly shuttled to cellular hot spots where, most likely, its actin-crosslinking or protein-scaffolding functions are needed. On the other hand, CCL2-stimulated CCR2B was seen travelling in vesicles along filamin A-containing actin filaments that concentrated around the perinuclear region. It is well know that late endosomes localize in those areas, but it is not so clear that the filamin A protein through its binding to actin filaments is required for bringing the CCR2B-endosomes there.

Both endosomes and endogenous vesicles can be propelled through the cytoplasm by actin comet tails and this process has been described for several vertebrate species [34]. Although the exact function of these structures has not been thoroughly characterized, it has been proposed that comet tails might facilitate the movement of organelles towards cytoskeletal structures or aid the fusion of endocytic vesicles [34]. Active actin-filament assembly seems to provide the motor for pushing the endosomes along and both Arp2/3 (actin-related protein 2/3) complexes [34] and the large GTPase dynamin (Dyn2) [35] have been described as components of actin comet tails. Furthermore actin dynamics are controlled by RhoB and mDia, both localized in endosomes, which contribute to the process of vesicle movement [36]. Due to its multitude of interaction partners, it is appropriate to assume that during this vesicle transport, FLNa is associated with signalling molecules important for RhoGTPase-mediated cytoskeletal remodelling. It has been shown before that FLNa interacts with proteins such as the actin-regulating Ras-related small GTPases RhoA, Rac1, Cdc42 and RalA [15], the guanine nucleotide-exchange factor (GEF) for Rac1 and RhoG, Trio [37], the RhoGEF Lbc [38] and the RhoGTPase effectors Pak1 [39], ROCK [40] and FilGAP [41]. It is also known that FLNa associates dynamically with Golgi membranes during budding and trafficking of transport vesicles and has been shown that in periventricular nodular heterotopia, a developmental FLNa loss-of-function disease, this vesicle transport is disrupted [42], [43]. This suggests that FLNa not only functions as a structural protein, but also, that it has an important role as a cargo protein for the transport of components to dynamic cellular locations. Therefore, taken together our findings suggest that one of the functions of FLNa is to transport vesicles with the stimulated chemokine receptor to these dynamic cellular locations where it will redistribute it. Particularly interesting is the finding that some of the vesicles with CCL2-stimulated CCR2B headed towards the leading edge of the cell.

In conclusion, these findings open up new opportunities to use this tool for investigating and characterizing the potential interaction of FLNa with chemokine receptors and its role in the vesicular trafficking.

## Materials and Methods

### Cell culture

Human melanoma cell line M2 (lacking expression of FLNa) and isogenic cell line A7 (stably expressing full-length FLNa) (provided by J. Hartwig, Harvard Medical School, Boston, MA, USA) were maintained in α-minimal essential medium (MEM) supplemented with 8% (v/v) newborn calf serum, 2% (v/v) fetal calf serum (FBS) [23]. A7 and M2-FLNa-EGFP cells were cultured in the presence of 500 μg/ml G418. Cells were maintained in a humidified atmosphere of 5% CO_2_ at 37°C. FLAG-CCR2B expressing HEK293-stable and HEK293 cells were grown in Dulbecco's modified Eagle's medium (DMEM) supplemented with 10% (v/v) FBS and 250 µg/ml G418.

### DNA Constructs

The pcDNA3.1-FLNa-EGFP vector was derived from pREP4-FLNa (provided by J. Hartwig, Harvard Medical School, Boston, MA, USA) and pEGFP-N1 (Clontech, Sant-Germain-en-Laye, France) using PCR. The FLNa sequence from pREP4-FLNa was amplified in two pieces resulting in an N-terminal fragment (aa 1–1742) and a C-terminal fragment (aa 1743–2647). The EGFP fragment was amplified from the pEGFP-N1 and subsequently inserted in between the N-terminal and C-terminal FLNa fragments in a pcDNA3.1 vector. The FLNa N-terminal fragment was amplified utilizing the forward primer: 5′CTC GCT AGC CTG AAA ATG AGT AGC TCC CAC, and reverse: 5′CCC AAG CTT CAG AGC CAG AGC CG. The C-terminal fragment was amplified utilizing the forward primer 5′CCC AAG CTT CTC GGG GAC CAG CCC TC, and reverse: 5′GAG GCG GCC GCT CAG GGC ACC ACA AC. For the EGFP fragment, forward: 5′GGA AAG CTT GCG ATG GTG AGC AAG GGC, and reverse: 5′CCG AAG CTT GGA CTT GTA CAG CTC GTC. Sequencing was performed in order to determine the correct insertion of EGFP in the hinge 1 region of FLNa. pCherry-Rab-5 was derived from Rab5-GFP (provided by P. van der Sluijs, University Medical Center Utrecht, The Netherlands) using PCR and cloning into pCherry-C1 (Clontech Laboratories, Inc, USA) in XhoI and HindIII. The Flag-tagged ß2-adrenergic receptor in pcDNA was a kind gift of F. Mayor (Centro de Biologia Molecular, Madrid, Spain).

### FLNa-EGFP homology modeling structure

To estimate the 3D structure of the generated FLNa-EGFP hinge 1 region, an automated homology modeling prediction was calculated by uploading the target sequence to the Swiss-model server (SIB, Lausanne, Switzerland).

### Establishment of stable clones

M2 cells were transiently transfected with pcDNA3.1-FLNa-EGFP using Fugene 6 (Roche Diagnostics, Basel, Switzerland) according to the manufacturer's instructions. After two days, the culture medium was replaced with medium containing G418 (1000 μg/ml). Resistant colonies were sorted twice using a BD FACSAria SORP flow cytometer to select for cells expressing high fluorescence. From the mixed population, cells were serially diluted into multiwell plates and nine clones (C1–C9) were picked and cultured separately. Cells were further cultured in medium containing 500 μg/ml G418.

### Immunoblotting

For checking the expression of the chimera, A7 and M2 cells were transiently transfected with pcDNA3.1-FLNa-EGFP or pcDNA3.1 using Fugene 6 according to the manufacturer's instructions. After 48 h, cells were washed in ice-cold 1x PBS, before being solubilized in 0.5% NP-40 lysis buffer (50 mM Tris, pH 7.4, 150 mM NaCl, and 0.5% NP-40) containing 1 mM EDTA, 0.1 mM Na_3_VO_4_ and a cocktail of protease inhibitors. After clarification, the lysates were resolved on SDS-PAGE and transferred to nitrocellulose membranes. The presence of FLNa and FLNa-EGFP was detected using anti-filamin 1 (Santa Cruz Biotechnologies, CA, USA) and anti-GFP (Abcam, Cambridge, UK) antibodies. For assessing the expression levels of FLNa-EGFP in the isolated stable cell clones, the same number of A7, M2 and M2-FLNa-EGFP cells were seeded in 100 mm plates. Twenty-four hours after plating, cells were washed, lysed and subjected to immunoblotting as described before. The presence FLNa, FLNa-EGFP and β-actin was detected using anti-filamin 1 and anti-β-actin (Abcam) antibodies. Blots were developed using an enhanced chemiluminescent method (Pierce, Thermo Scientific, Rockford, USA) and scanned using the LAS-3000 imaging system (Fujifilm, Tokyo, Japan).

### Scanning electron microscopy (SEM) and correlational microscopy

Scanning electron microscopy for the structural characterization of M2, A7 and M2-FLNa-EGFP cells was performed as follows: after dehydration in graded alcohols, cells seeded on top of coverslips were dried by the critical point method, mounted on cylinders with double-coated adhesive tape and covered with a thin layer of gold/palladium in an anion sputter coating unit. Scanning electron microscopy was carried out using a Jeol JSM-7400F scanning electron microscope (Jeol ltd. Tokyo, Japan) at an acceleration voltage of 5.0 kV and a working distance of 7.7 mm in high-vacuum mode. For the correlational microscopy, samples used for the SEM were first pictured with a Nikon TE2000 inverted fluorescent microscope (Nikon, Tokyo, Japan), using marked positions in the coverslip in order to identify the cells after the SEM treatment. Finally, SEM and widefield fluorescent images were overlapped employing the graphic software package Adobe PhotoShop CS4.

### Wound healing assay

Monolayers of A7, M2, C4 or C5 cells were wounded 24 h after plating in a 12-well plate by scratching with a 200 μl pipette tip. Debris was removed by washing. Images of the wound were taken at 4× magnification (Nikon PhL; NA 0.13) at times 0, 24 and 48 h after wounding using a NikonTE2000 inverted fluorescence microscope equipped with a MuTech MV 1500 camera (Nikon Instruments Europe B.V., The Netherlands). Wound closure was quantified using the Image J software by measuring the total size of each wound area at different time points.

### Immunostaining and confocal microscopy

To analyze the subcellular localization of FLNa-EGFP, A7 and M2 cells were seeded on coverslips and M2 cells were transiently transfected with pcDNA3.1-FLNa-EGFP or pREP4-FLNa using Fugene 6. Cells were then washed in PBS, fixed and permeabilized in PBS with 0.1% Tween 20 before staining with mouse anti-filamin 1 (Santa Cruz Biotechnologies, CA, USA), Alexa Fluor 488 goat anti-mouse (Invitrogen, CA, USA) or Texas Red goat anti-mouse (Invitrogen, CA, USA) and Rhodamine-Phalloidin (Invitrogen, CA, USA). All coverslips were mounted using Prolong Gold mounting medium with DAPI (Invitrogen, CA, USA). Optical sections were acquired using a Leica TCS SP2 AOBS confocal system equipped with a 63x Ibd.BL oil objective. Colocalization analyses were performed using the Imaris colocalization module (Bitplane AG, Zurich, Switzerland).

### Live cell imaging

For long term live cell imaging, a TCS SP2 and SP5 confocal microscopes (Leica Microsystems) equipped with a 37°C incubation chamber with 5% CO2 were used. For mitosis, wound-healing and receptor internalization, cells were stimulated with 40 nM CCL2. ß2-AR-transfected HEK293 cells were stimulated with 10 µM of isoproterenol. After adding of the ligand, four slice Z-stacks were taken every 2 min for 14 h for mitosis and wound-healing experiments. The collected images from the time-lapse imaging were processed using Imaris (Bitplane) and mounted as .AVI files with a frame rate of 14 fr/sec. For short-term live imaging, a Perkin-Elmer Ultraview spinning wheel confocal system mounted on a Zeiss Axiovert 200 microscope and equipped an Orca-ER Firewire Camera CCD camera (Hamamatsu, Hamamatsu City, Japan) and with a 37°C incubation chamber with 5% CO2 was used. Z-stacks of the images were collected using a 63X NA 1.4 oil objective with Argon excitation laser at 488 nm and Helium-Neon Laser at 543 nm. HEK293 cells stably expressing FLAG-CCR2B were incubated during 1h at 4°C with anti-FLAG M2-Cy3 antibody (Sigma-Aldrich Co). Z-stacks were acquired continuously in the spinning disk with 300 millisecond exposures during 60 min after addition of 1 ml of 40 nM CCL2 on top of the plate with 1ml of DMEM medium without phenol red. Experiments for receptor internalization together with Cherry-Rab5 were done in a confocal SP5 microscope (Leica Microsystems) acquiring one plane (x/y/t) every two minutes time point. HEK293 cells were trasient transfected with pcDNA3-FLAG-CCR2B or FLAG-ß2AR in presence of Cherry-Rab5 and pcDNA3.1-FLNa-EGFP. The pinhole was set to 3 to diminish image bleaching and to increase acquisition speed. Green, red and far red channels were captured sequentially. Images were processed using Imaris (Bitplane AG) or Fiji software and mounted as .AVI file with a frame rate of 7 or 14 fr/sec. Colocalization analyses were performed using the Imaris colocalization module (Bitplane AG).

### Tracking analyses

Tracking analyses of the CCR2B trajectory were done using the 4D tracking module from Imaris software (Bitplane AG) uploading the z-stack captured by spinning disk, segmentating CCR2B receptors to red spheres and drawing the trajectories of movements of the red spheres. Tracking provided qualitative information regarding the fluorescence intensities of the CCR2B receptor (red balls), particle's trajectory and calculated displacements. The color coded diagram ranging from 0 min (purple) until 20 min (yellow) shows the kinetic of the receptor through time. Arrow depicts the leading edge of the cell.

### Adhesive micropatterning assay

In order to normalize the cell architecture for studying FLNa, A7, M2 and C5 cells were plated 4 h at 37°C on glass coverslips microprinted with fibronectin patterns (CYTOO, Grenoble, France). Cells were fixed, permeabilized and incubated with primary and secondary fluorescent antibodies as follows: for A7 and M2 cells, mouse anti-filamin 1, Alexa 488 goat anti-mouse (Invitrogen) and Rhodamine-phalloidin (Invitrogen) were used, while Rhodamine-Phalloidin was used for the C5 cells. Finally, samples were mounted using DAPI Prolong Gold mounting medium (Invitrogen) and optical sections were acquired using a Leica TCS SP2 AOBS confocal system. Colocalization analyses were performed using the Imaris colocalization module (Bitplane). To quantify the rigidity of the cells with or without FLNa, the imageJ (NIH) software was used to measure the area in pixels of the cell attached to the pattern and the area in pixels of the fibronectin pattern. The ratio of rigidity was calculated dividing both values from 30 random cells per type and shape and plotting the average results (±SEM) in a chart.

### CCR2B internalization assay in presence of FLNa siRNA

A synthetic FLNa-targeted siRNA oligoribonucleotide duplex with symmetric 3′TT overhang (FLNa siRNA sense sequence: 5′-AGGUGCUGCCUACUCAUGA-3′, [44]) was purchased from Dharmacon RNA Technologies (USA, Lafayette, CO). The following two oligoribonucleotides were used as negative control: siGENOME negative control siRNA #3 (Dharmacon), AllStars negative control siRNA (Qiagen). HEK293 cells stably expressing FLAG-CCR2B were transiently transfected with 100 nM synthetic control siRNA or FLNa siRNA using Lipofectamine^TM^ 2000 reagent (Invitrogen), and incubated 5 days at 37°C [17]. Cells were then washed in ice-cold PBS before being solubilized in 0.5% NP-40 lysis buffer (50 mM Tris, pH 7.4, 150 mM NaCl, and 0.5% NP-40) containing 1 mM EDTA, 0.1 mM Na_3_VO_4_ and a cocktail of protease inhibitors. After clarification, the lysates were resolved on SDS-PAGE and transferred to nitrocellulose membranes. Cells to be used for the internalization assay were trypsinised 4 days after transfection and replated onto poly-L-lysine-coated glass coverslips. After 24 h, cells were incubated with mouse anti-FLAG-M2-Cy3 antibody (Sigma-Aldrich Co.) at 4°C and stimulated with 20 nM CCL2 at 37°C for the times indicated before being fixed, and incubated with goat anti-FLNa (Santa Cruz Biotechnology, Inc.) followed by FITC-conjugated mouse anti-goat (Jackson ImmunoResearch, Suffolk, UK).

## Supporting Information

Figure S1 (**A**) Immunofluorescence images of the cell morphology and expression pattern of FLNa-EGFP in three selected M2 clones (C5, C8 and C4) stably expressing FLNa-EGFP. Images are superposed on top of the bright-field images taken from the same area of the plates (magnification 20x). (**B**) Immunoblot showing the levels of FLNa-EGFP in stable M2 clones C5, C8 and C4 versus the levels of endogenous FLNa in A7 cells. M2 cells were used as control. β-actin bands were used as a loading control. Experiments were repeated twice with similar results.
(TIF)

Figure S2 Behavior of FLNa-EGFP in migration. Time-lapse images from M2, A7 and M2 FLNa-EGFP cells from selected areas showing the directional migration of the cells during 4 h. Dotted lines mark the boundaries of the cell. The monochromatic images are a segmentation from the bright-field images showing one single cells per case (magnification 40x). Scalebar (10 nm).
(TIF)

Figure S3 Immunoblot quantification of FLNa knockdown in HEK293-CCR2B cells treated with 100 nM siRNA as indicated. Histogram represents the intensity of the FLNa bands, normalized against the corresponding ß-actin bands. Experiments were repeated twice with triplicate samples.
(TIF)

Figure S4 Movie from the SP5 Leica confocal images showing a HEK293 transfected cell expressing FLNa-EGFP, Cherry-Rab5 and Flag-CCR2B labeled M1 with anti-flag antibody and secondary Alexa 647 (shown in blue). The film shows the internalization of Flag-CCR2B (blue) after CCL2 stimulation and the co-localization with Rab 5 (red) during 30 min. The film is from one single layer of the Z stacks and has been at converted to 1 frame every 5 seconds. Experiments were repeated three times.
(AVI)

Figure S5 Time lapse of the internalization of the isoproterenol-stimulated ß-2AR in HEK293 cells expressing Cherry-Rab5 and FLNa-EGFP. HEK293 cells were transfected with pcDNA-ß-2AR, pCherry-Rab5 and pcDNA3.1-FLNa-EGFP. Cells were incubated with anti-flag M1 antibody followed by anti-mouse-Alexa647 antibody at 4°C. Cells were place in the 37°C microscopy chamber and stimulated with 10 mM isoproterenol. Snapshot from Leica confocal SP5 time lapse experiments with isoproterenol-stimulated HEK293 cells at 3:56 min showing the distribution of ß-2AR (blue), Cherry-Rab5 (red) and FLNa-EGFP (green) (upper panel). Lower panels show the snapshots of different time points (3∶56 to 4∶28 min) with separate channels.
(TIF)
