# Supplementary figures and images for: Filamin A-Hinge Region 1-EGFP: A Novel Tool for Tracking the Cellular Functions of Filamin A in Real-Time
Source: PLoS One. 2012 Aug 1;7(8):e40864. doi: 10.1371/journal.pone.0040864 (PMC3411599; doi:10.1371/journal.pone.0040864)

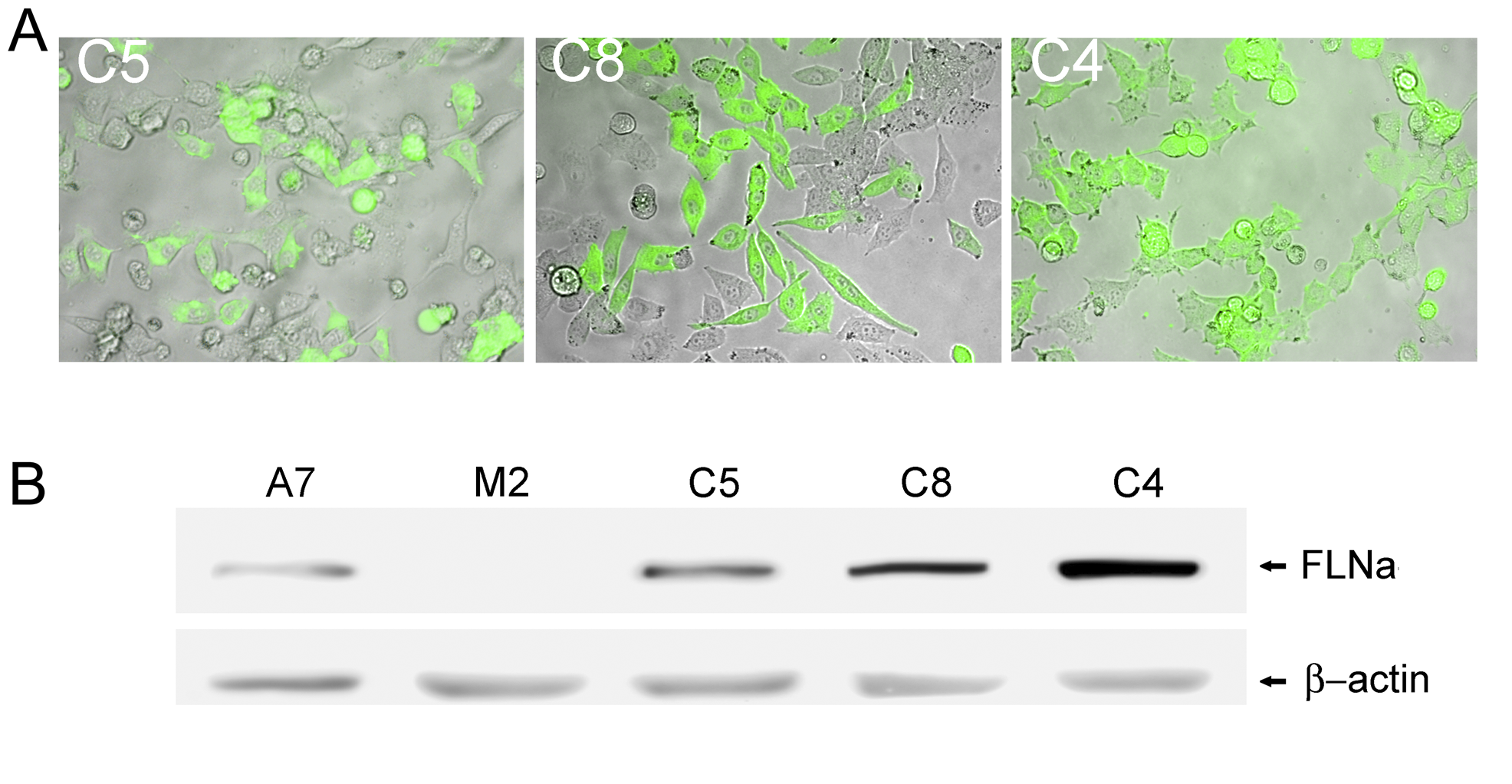

Supplement: Figure S1 — (A) Immunofluorescence images of the cell morphology and expression pattern of FLNa-EGFP in three selected M2 clones (C5, C8 and C4) stably expressing FLNa-EGFP. Images are superposed on top of the bright-field images taken from the same area of the plates (magnification 20x). (B) Immunoblot showing the levels of FLNa-EGFP in stable M2 clones C5, C8 and C4 versus the levels of endogenous FLNa in A7 cells. M2 cells were used as control. β-actin bands were used as a loading control. Experiments were repeated twice with similar results. (TIF) [file pone.0040864.s001.tif]

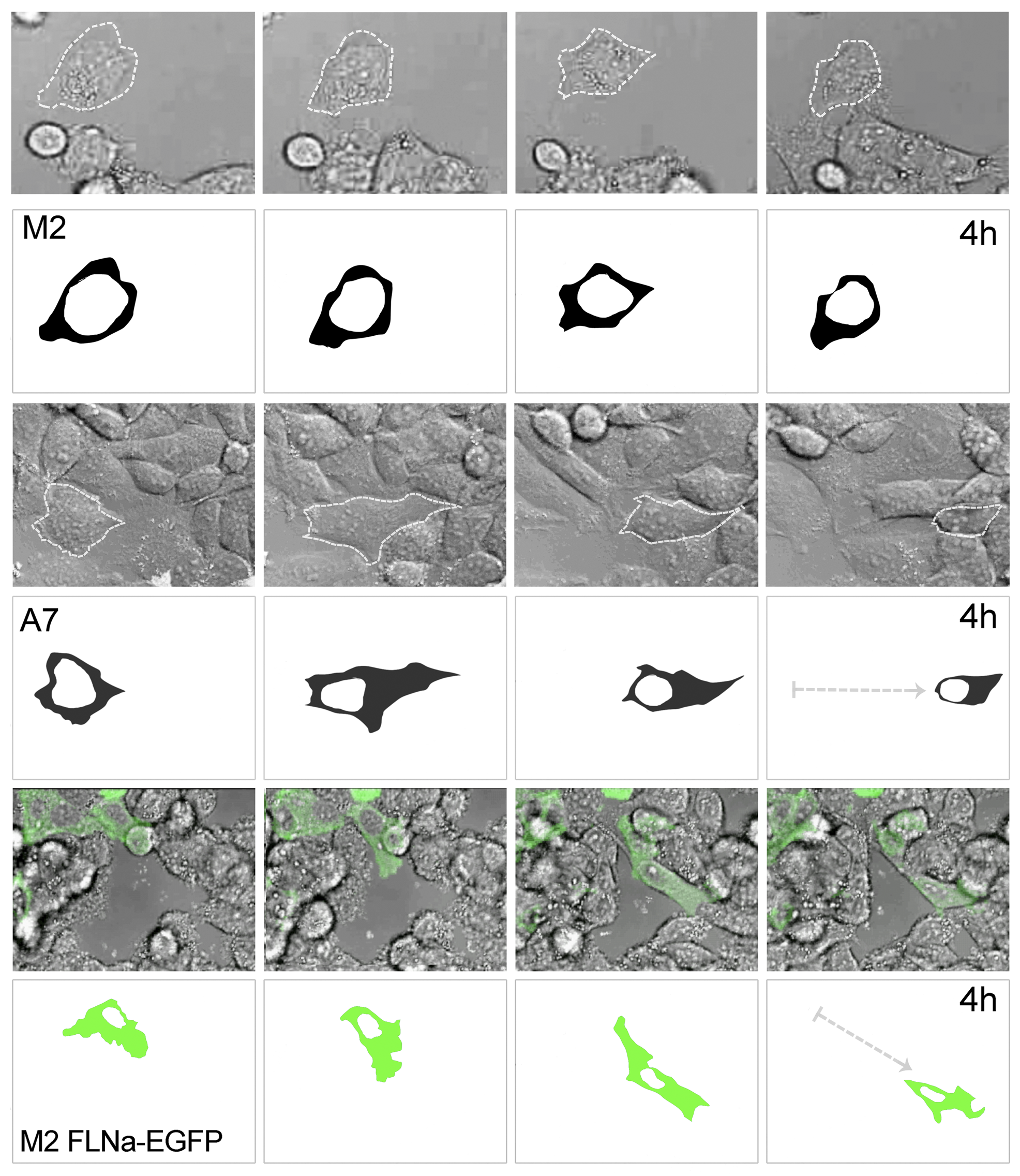

Supplement: Figure S2 — Behavior of FLNa-EGFP in migration. Time-lapse images from M2, A7 and M2 FLNa-EGFP cells from selected areas showing the directional migration of the cells during 4 h. Dotted lines mark the boundaries of the cell. The monochromatic images are a segmentation from the bright-field images showing one single cells per case (magnification 40x). Scalebar (10 nm). (TIF) [file pone.0040864.s002.tif]

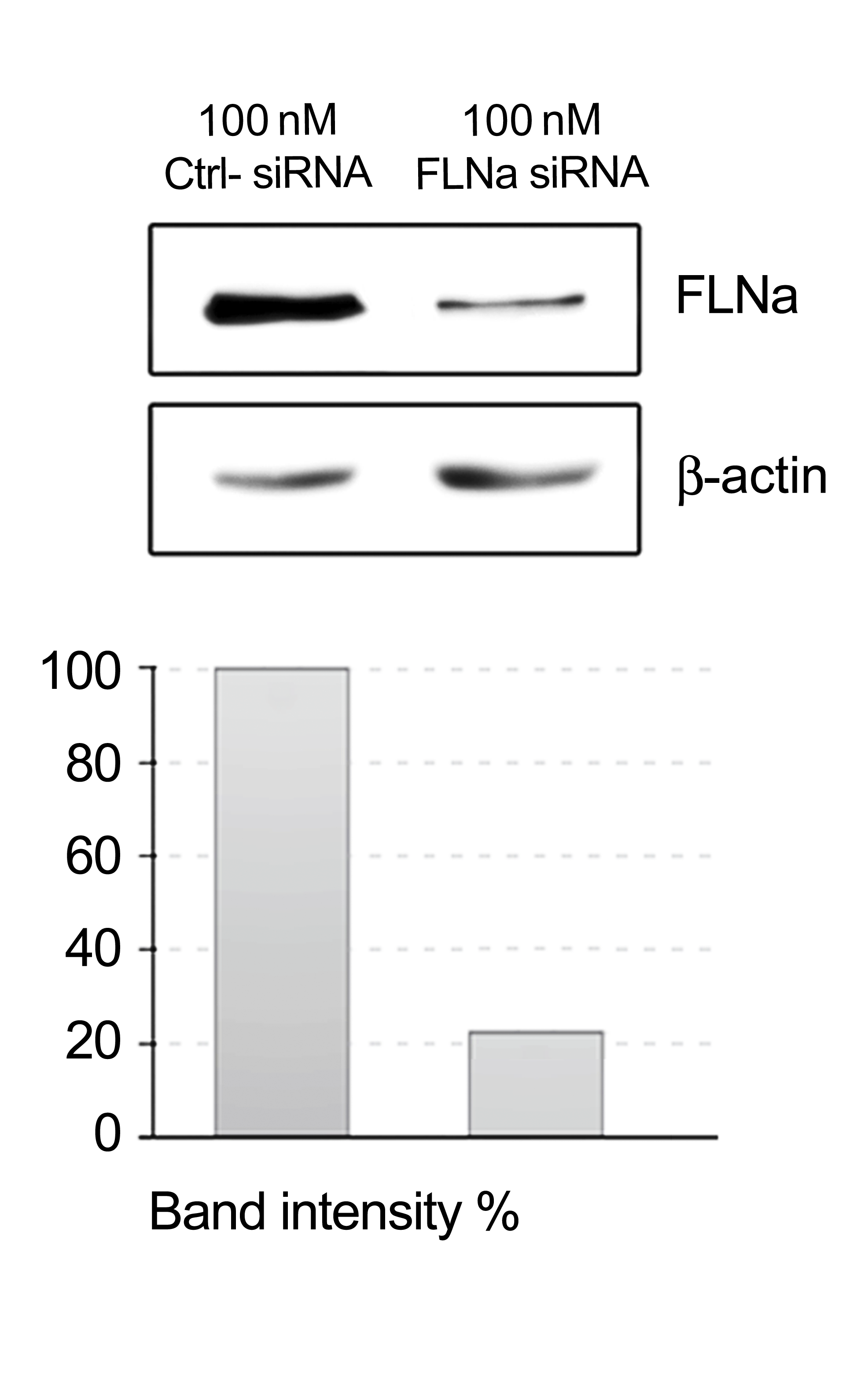

Supplement: Figure S3 — Immunoblot quantification of FLNa knockdown in HEK293-CCR2B cells treated with 100 nM siRNA as indicated. Histogram represents the intensity of the FLNa bands, normalized against the corresponding ß-actin bands. Experiments were repeated twice with triplicate samples. (TIF) [file pone.0040864.s003.tif]

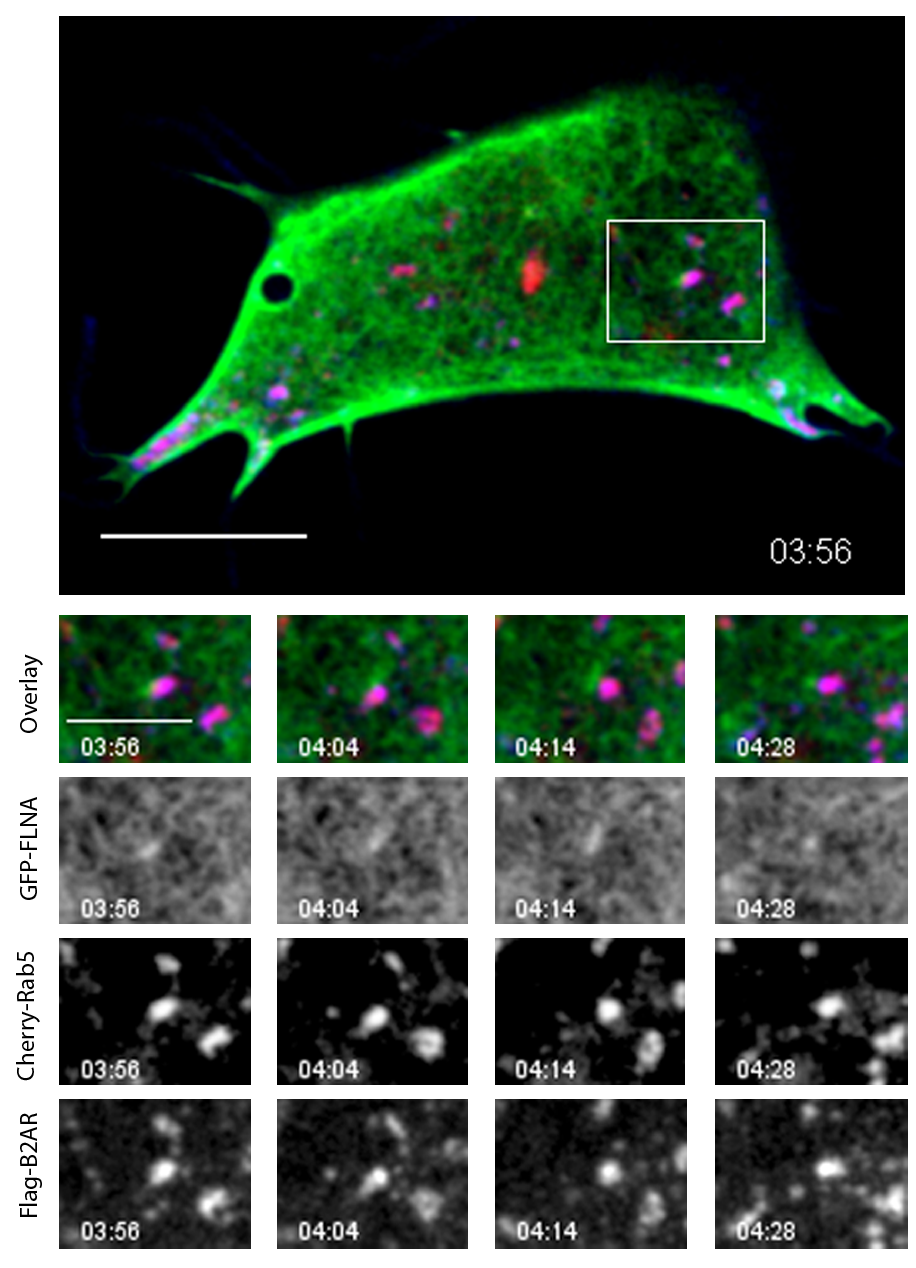

Supplement: Figure S5 — Time lapse of the internalization of the isoproterenol-stimulated ß-2AR in HEK293 cells expressing Cherry-Rab5 and FLNa-EGFP. HEK293 cells were transfected with pcDNA-ß-2AR, pCherry-Rab5 and pcDNA3.1-FLNa-EGFP. Cells were incubated with anti-flag M1 antibody followed by anti-mouse-Alexa647 antibody at 4°C. Cells were place in the 37°C microscopy chamber and stimulated with 10 mM isoproterenol. Snapshot from Leica confocal SP5 time lapse experiments with isoproterenol-stimulated HEK293 cells at 3:56 min showing the distribution of ß-2AR (blue), Cherry-Rab5 (red) and FLNa-EGFP (green) (upper panel). Lower panels show the snapshots of different time points (3∶56 to 4∶28 min) with separate channels. (TIF) [file pone.0040864.s005.tif]
